# Early weaning in pregnant cows as a fetal programming strategy alters the muscle metabotype and carcass traits in Nelore cattle

**DOI:** 10.1007/s11250-026-05237-w

**Published:** 2026-08-04

**Authors:** Matheus Sousa de Paula Carlis, Gabriela Abitante, Thiago Kan Nishimura, Carl Robertson Dahlen, Nara Regina Brandão Cônsolo, Luiz Alberto Colnago, Fernanda Maria Marins Ocampos, Germán Darío Ramírez-Zamudio, Patricia Maloso Ramos, Saulo Luz Silva, Miguel Henrique de Almeida Santana, Arlindo Saran Netto, Guilherme Pugliesi, Paulo Roberto Leme, Rodrigo Silva Goulart

**Affiliations:** 1 Department of Animal Science, School of Animal Science and Food Engineering, University of São Paulo, Pirassununga, São Paulo 13635-900 Brazil; 2 Department of Animal Reproduction, School of Veterinary Medicine, and Animal Science, University of São Paulo, Pirassununga, São Paulo 13635-900 Brazil; 3 Department of Animal Science, Center for Nutrition and Pregnancy, North Dakota State University, NDSU Department 7630, Fargo, ND 58108-6050 USA; 4 Department of Animal Nutrition and Production, Faculty of Veterinary Medicine and Animal Science, University of São Paulo, Pirassununga, SP 13635-900 Brazil; 5 Embrapa Instrumentation, Brazilian Agricultural Research Corporation (Embrapa), São Carlos, SP 13561-206 Brazil

**Keywords:** Fetal programming, Maternal nutrition, Muscle metabolomics, One-carbon metabolism, Retail cuts yield

## Abstract

This study evaluated early weaning (EW) versus conventional weaning (CW) and dam parity (DP) effects on carcass traits, meat quality, and the muscle metabolome of male offspring exposed in utero during the intervention. A randomized complete-block design with a 2 × 2 factorial arrangement compared weaning strategies (WS: EW at 150 d lactation/80 d gestation vs. CW at 240 d lactation/170 d gestation) and dam parities (DP: multiparous [MP] vs. secundiparous [SP]). Carcass and meat quality data were analyzed considering the dams as experimental unit by using the Mixed procedure, considering the WS and DP as fixed effect, and their interaction. There was interaction between WS×DP (*P <* 0.01) for hindquarter fat trim where EW-MP and CW-MP carcasses required more trim fat than EW-SP. EW bulls tended to reduce drip loss (*P =* 0.07), tended to increased pistola hindquarter (% of carcass; *P =* 0.09), while significantly raising ultimate carcass pH (*P* = 0.04) versus CW. An untarget ^1^H NMR-based metabolomic approach was applied to detect metabolic changes. Pathway analysis of differentially abundant metabolites revealed the upregulation of betaine and glycerolipid metabolism, methylhistidine metabolism for WS and homocysteine degradation, phosphatidylethanolamine synthesis and selenoamino acid metabolism for DP. Offspring of MP dams had heavier cold carcasses (*P =* 0.03), greater Forequarter, brisket, shank (*P <* 0.03), smaller pistola hindquarter and top sirloin cap (*P ≤* 0.05) than those of SP. These results indicate that WS and DP induce metabolomic shifts favoring muscular hypertrophy and one-carbon metabolism, which may enhance muscularity without impairing meat quality.

**Supplementary Information:**

The online version contains supplementary material available at https://doi.org/10.1007/s11250-026-05237-w.

## Introduction

In Brazil’s extensive pasture systems, beef cows typically carry pregnancies through the dry season, when both forage quantity and quality are limited, placing lactating dams in a severe negative energy balance as they must simultaneously support fetal growth and milk production (Costa et al. 2021; Gionbelli et al. 2024). This challenge is exacerbated in young, primiparous, and secundiparous cows, which still demand nutrients toward their own growth (Costa e Silva et al. 2015; NASEM, 2016). Because skeletal muscle tissue has a lower priority in nutrient partitioning compared to vital organs, maternal undernutrition during the critical windows of secondary myogenesis (mid- to late-gestation) can permanently compromise fetal muscle development and metabolic function (Zhu et al. 2006; Hocquette 2010; Du et al. 2010a).

In this scenario, the early weaning (EW) has emerged as a management strategy to alleviate maternal nutrient drain during mid-gestation by curtailing lactation, thereby redirecting the nutrients toward the developing fetus (Oattes et al. 2021). In *Bos indicus*, EW has been shown to enhance dam body condition, elevate calf birth weights and improve reproductive performance in the next breeding season (Nishimura et al. 2023a, b; Oattes et al. 2021). However, its implications for *Bos indicus* cows and, critically, the resulting carcass composition, meat quality, and underlying muscle metabolomics profiles of their offspring have not been thoroughly investigated.

Studies have demonstrated that enhancing maternal nutrition, either throughout gestation or specifically during late gestation, via protein supplementation alters the abundance of key muscle and adipose metabolites in Nelore cattle. Fernandes et al. (2024) reported increases in glycerophospholipids, amino acids, sphingolipids, biogenic amines, phosphatidylcholine, and lysophosphatidylcholine in both *Longissimus thoracis* and subcutaneous fat of progeny from supplemented dams. Likewise, Schalch Junior et al. (2022) showed that improved maternal diet elevates plasma concentrations of metabolites involved in protein metabolism in both dams and their offspring. Building on this integrative “-omics” approach, Polizel et al. (2025) combined plasma metabolomics, fecal microbiome, and ruminal fluid microbiome analyses to reveal that late-gestation protein supplementation enhances pathways related to protein turnover, lipid metabolism, and methane production in Nelore bulls.

This study complements our previous work Carlis et al. (2025a), which originated from the same research project. There, we reported complex effects of the EW strategy on feedlot performance and muscle gene expression in these animals. While EW bulls, particularly from secundiparous dams, showed compensatory performance improvements mitigating lower initial weights observed in some CW groups, they paradoxically tended to exhibit smaller ribeye areas (REA) and possessed muscle fibers with smaller cross-sectional area and diameter at slaughter compared to CW bulls. However, EW bulls displayed significantly greater muscle mRNA abundance of the eukaryotic translation initiation factor 4E (*EIF4E*) and exhibited a tendency toward increased expression of the mechanistic target of rapamycin kinase (*MTOR*), key regulators of protein synthesis. Furthermore, EW bulls tended to have greater levels of insulin serum concentrations at beginning of the feedlot period compared to CW group. This apparent discrepancy between the muscle phenotype at slaughter (smaller fibers/REA) and the underlying molecular signaling (upregulated protein synthesis pathways) suggests that programming via EW may sustain anabolic potential longer into the finishing phase or alter the trajectory of muscle development.

Therefore, understanding the muscle metabolome is crucial to elucidate the biochemical mechanisms driving these programmed effects of weaning strategy on muscle characteristics and overall carcass composition. Our hypothesis posits that early weaning induces persistent metabolic programming in male fetuses exposed in utero, specifically by shifting the muscle metabolome to favor pathways associated with enhanced protein synthesis capacity and modified energy substrate utilization. These effects would subsequently influence carcass composition (particularly relative muscle yields) without negatively impacting key meat quality attributes like tenderness and color. The primary objective of this research was to assess how maternal weaning strategies—specifically early weaning (150 days of lactation/80 days gestation) versus conventional weaning (240 days of lactation/170 days gestation)—interact with dam parity to influence the carcass characteristics, meat quality, and muscle metabolome of male offspring exposed in utero.

## Material and methods

The detailed experimental design, animal management, and dietary composition are fully described by Carlis et al. (2025a, b). This experiment was conducted at the School of Animal Science and Food Engineering (FZEA) of the University of São Paulo (USP), under the approval of the Commission on Ethics in Animal Use (CEUA: 2884250620).

### Experimental design and feeding

**Fig. 1:**
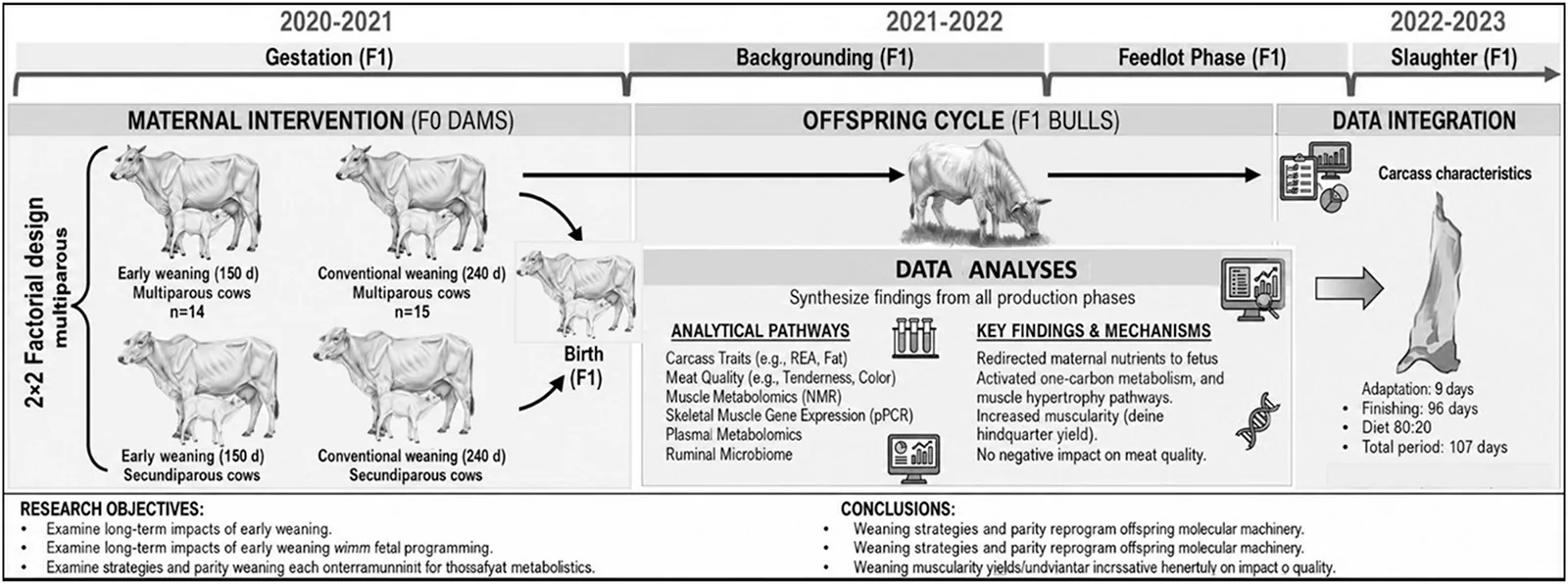
Timeline of experimental events, beginning with the birth of calves (first generation), which were weaned at either 150 d (80 d of gestation) or 240 d (170 d of gestation) across different parity groups of cows (multiparous or secundiparous). All cows were managed within five management blocks during the gestation and suckling phases. Following weaning at 150 d of lactation, the calves were managed within the same management group during the background phase. On the feedlot phase the bulls were sorted in collective pens (*n* = 3) by initial BW, weaning strategy, and Dam parity

**Fig. 2:**
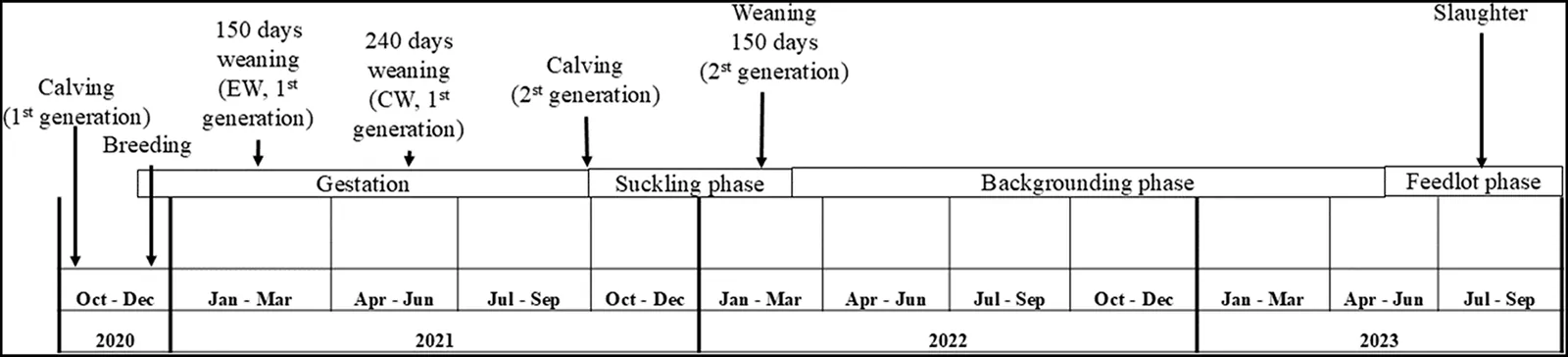
Schematic diagram of experimental design, animal management, and production timeline. The study utilized a randomized complete-block design in a 2 × 2 factorial arrangement to evaluate the effects of maternal weaning strategy and dam parity on male offspring (in utero during the intervention). Male calves (F1) were managed as a single group during the backgrounding phase and subsequently housed in collective pens during the feedlot phase until slaughter

The experiment was designed (Figs. 1 and 2) and was fully described by Carlis et al. (2025a, b). Briefly, 58 pregnant dams were allocated to the treatments. However, two male calves were lost during the cow-calf and backgrounding phases due to reasons unrelated to the experimental treatments. Consequently, a total of 56 male offspring completed the feedlot phase and were evaluated at slaughter. The experiment was analyzed as a randomized complete block in a 2 × 2 factorial arrangement with two weaning strategies (early weaning [EW, *n* = 27] or conventional weaning [CW, *n* = 29]), and two dam parities (multiparous dams [MP, *n* = 29] or secundiparous dams [SP, *n* = 27]). This resulted in the following combinations: EW-MP: early weaning in multiparous dams (dams whose calves have been weaned at 150 days of lactation or 80 days of gestation; *n* = 15); CW-MP: conventional weaning in multiparous dams (dams whose calves have been weaned at 240 days of lactation or 170 days of gestation; *n* = 14); EW-SP: early weaning in secundiparous dams (dams whose calves have been weaned at150 days of lactation or 80 days of gestation; *n* = 12); CW-SP: conventional weaning in secundiparous dams (dams whose calves have been weaned at 240 days of lactation or 170 days of gestation; *n* = 15).

The bulls evaluated in this study were fetuses in utero during the application of maternal treatments. A comprehensive description of the experimental design, encompassing the entire production cycle (cow-calf, backgrounding, and feedlot phases), dietary management, and performance data, is provided in Carlis et al. (2025a, b). For brevity and to avoid textual repetition regarding the experimental design and animal management already published in our companion papers (Carlis et al. 2025a, b), only the methodologies specific to carcass traits and meat quality assessment are detailed herein.

Male offspring (*n* = 56) from these dams were identified at birth, remained with their mothers until their respective weaning dates, and were subsequently backgrounded as a single group until feedlot entry. The bulls were transferred to the experimental feedlot at 18 ± 0.9 months of age and an average body weight (after fasting) of 413.5 ± 23.23 kg. At the begin of feedlot, the bulls received a deworming and immunomodulation, utilizing 1 ml/ 20 kg of body weight (BW) Levamisole Phosphate (Ripercol^®^, Zoetis, Campinas, SP, Brazil) and 10 ml/ animal Albendazole Sulfoxide (Agenbendazol^®^, Agener União Saúde Animal, São Paulo, SP, Brazil) for deworming. Additionally, the bulls received radio frequency identification (RFID) electronic ear tags to evaluate individual feed intake and behavior. At the beginning of the feedlot phase, the 56 bulls were stratified by the main factor combinations (EW-MP, CW-MP, EW-SP, CW-SP) and ranked by their initial fasting body weight. To ensure a balanced experimental design, the animals within each stratum were systematically allocated across three blocks and subsequently housed in three collective pens to equalize the average initial body weight. Upon feedlot entry, the bulls were equipped with electronic feeders and waterers (Intergado^®^, Ponta Inc., Betim, MG, Brazil). To ensure the efficacy of this allocation procedure, the initial fasting body weight was statistically evaluated prior to the trial. This analysis confirmed the absence of statistical differences among treatments within each block, with significant variations occurring exclusively between the blocks, thereby validating the homogeneity of the experimental starting conditions.

Experimental diets (Table 1) were formulated using RLM software (Integra Software, Piracicaba, São Paulo, Brazil) to meet the nutrient requirements of Nellore bulls gaining 1.5 kg per day of body weight (BW) for a 98 ± 8 day finishing period.

**Table 1:** Ingredients and chemical composition of total mixed ration during finishing phase

Item	Finishing diet
*Ingredient*,* % of DM*	
Corn silage	20.1
Ground Corn	70.0
Soybean meal	6.9
Urea	1.2
Mineral supplement^1^	0.4
Salt	0.4
Limestone	0.6
Potassium chloride	0.3
Magnesium oxide	0.04
*Chemical composition*,* % DM*	
Dry matter, % *As-fed*	59.1
Crude protein	14.8
Ethereal extract	3.5
Neutral detergent fiber	31.7
Acid detergent fiber	13.0
Ash	3.5
Total digestible nutrients	79.9

^1^Mineral supplement included: 1.0% trace mineral premix containing 230 g Ca, 160 g P, 60 g S, 160 mg Co, 160 mg Cu, 135 mg I, 2.700 mg Mn, 8.100 mg Zn, and 4.000 mg of monensin sodium

### Slaughter and carcass traits

Bulls were slaughtered after 16 h of fasting at 21.8 ± 0.95 months of age with an average live weight of 569.1 ± 45.74 kg. Bulls were selected for slaughter within each block based on achieving a target of 3 mm subcutaneous fat thickness measured by ultrasound. Bulls were slaughtered in 4 groups at 7-day intervals at the University of São Paulo Abattoir, located 300 m from the feedlot facilities, minimizing pre-slaughter transport stress. Humane slaughter practices were followed as stipulated in the Regulations for Sanitary Inspection and Industrialization of Animal Origin Products (Brasil, 2017).

After removing the skin, head, feet, and viscera, the hot carcass weight (HCW) was recorded. Subsequently, the kidney, pelvic + inguinal fat weights were recorded during slaughter. After 24 h chill, the carcasses were weighed to obtain the cold carcass weight (CCW), and the cold carcass yield and drip loss were calculated.

The pH and temperature of the carcasses were measured 1, 3, 6, 9, and 24 h after slaughter in the *Longissimus thoracis* (LT) muscle at the 12th rib level, using a digital thermometer (Incoterm, Porto Alegre, RS, Brazil) and a pH meter equipped with a penetration probe (model HI8314, Hanna Instruments, São Paulo, SP, Brazil). The pH meter was calibrated before each measurement session using standard buffers (pH 4.0 and 7.0) with automatic temperature compensation (25 °C). The left half of each carcass was separated into the following wholesale cuts: Pistola hindquarter, Forequarter, and a combined section containing the brisket, short ribs, and flank. Kidney and flank fat were also separated and weighed. Subsequently, the wholesale cuts were dissected into retail cuts, bones, and trimmings. Retail cuts were standardized by trimming external fat to approximately 5 mm. Finally, total retail cuts were calculated as a percentage of carcass also as % of hindquarter. The hindquarter retail cuts were dissected following the guidelines by Standard, U. N. E. C. E. (2023).

### Meat quality and composition

Twenty-four hours after chill, three 2.50 cm-thick steaks were cut from the LT muscle between the 10th and 12th thoracic vertebrae of the left half of the carcass. Immediately, the steaks were individually vacuum-packed and were refrigerated at 2 °C for 1, 7, 14, and 21 days after slaughter. The steaks were analyzed to assess the meat color, cooking loss (CL), and Warner-Bratzler shear force (WBSF).

To measure the color, samples were removed from the vacuum packages, and allowed to bloom for 30-min and then L*, a*, and b* values were measured on the surface of the steaks, at three different points, using a spectrophotometric colorimeter (CM2500d, Konica Minolta Brazil, São Paulo, SP, Brazil), with a 10 mm aperture size, illuminant A, and 10° observer angle (AMSA 2012).

Cooking loss and WBSF were evaluated following the methodology outlined by the American Meat Science Association (AMSA 1995). Initially, the steaks were weighed to determine their initial weight. Then, a thermometer was inserted into the geometric center of each sample, which was subsequently heated in an industrial electric oven (model F130/L, Fornos Elétricos Flecha de Ouro Ind. e Com. Ltda., São Paulo, SP, Brazil) at 170 °C until the internal temperature of the samples reached 40 °C. After reaching the desired temperature, the samples were flipped and allowed to continue cooking in the oven until the internal temperature reached 71 °C. Subsequently, the samples were removed from the oven, cooled to room temperature (≅ 22 °C), and weighed. The cooking loss value was obtained as a percentage using the following formula: CL, % = ((Initial weight - final weight) / initial weight) × 100.

After weighing, the steaks were wrapped in plastic film and refrigerated at 4 to 6 °C for 12 h. To evaluate WBSF, six cores with a diameter of 1.27 cm, parallel to the orientation of the muscle fibers, were collected using a bench drill. Subsequently, these cores were subjected to a shearing test at a 200 mm/min rate using a Warner-Bratzler blade attached to a texture analyzer (TMS-PRO, Food Technology Corporation, Sterling, VA, USA). The WBSF was determined as the average of values obtained from the six cylindrical samples, and the results were expressed in Newtons (N).

The samples used for the visible/near infrared (Vis-NIRS) spectroscopic analysis were frozen after 1 day of chilling at 2 °C. The samples were thawed at room temperature (≅ 22 °C) and coarsely ground to fit into a circular glass cup (diameter 140 mm, depth 17.5 mm). The samples were analyzed using a near-infrared spectrometer (Food Scan, FOSS Electric A/S, Hillerød, Denmark) in transmittance mode to determine fat, moisture, protein, ash, short-chain fatty acids (SCFA), carbohydrates, water activity, energy, and Na (AOAC, 2007; #2007.04) for meat and meat products. The spectrometer operated at room temperature (≅ 22 °C) with a wavelength range from 850 to 1050 nm at 2 nm intervals. Each spectrum was an average of 24 sub-spectra recorded at 24 different points by automatically rotating the circular cup in the analyzer.

### Meat metabolomic profile

During slaughter, approximately 2 g of the *Longissimus thoracis* (LT) muscle were collected from the left side of the carcass, between the 12th and 13th thoracic vertebrae, before chilling. Samples were transferred to sterilized microtubes, snap-frozen in liquid nitrogen, and stored at − 80 °C until further analysis.

The metabolomic analysis was carried out following the methodology described by Cônsolo et al. (2020). Briefly, 0.5 g of frozen muscle tissue was homogenized using an Ultra-Turrax^®^ T25 Digital homogenizer (IKA, Campinas, SP, Brazil) in a chilled methanol: chloroform: water solution (2:2:1, v/v). The homogenates were vortexed for 1 min, incubated on ice for 15 min, and centrifuged at 10,000×g for 15 min at 4 °C. Supernatants were transferred to Eppendorf tubes and dried in a vacuum centrifuge (Itasul Import and Instrumental Technical Ltd.a, Porto Alegre, RS, Brazil).

The residues were resuspended in 550 µL of phosphate buffer prepared in D₂O containing 0.5 mM DSS-*d*₆ as the internal standard by Beckonert et al. (2007). Samples were transferred to 5 mm NMR tubes and analyzed on a Bruker Avance 14.1 T spectrometer equipped with a 5 mm Broadband Observe (BBO) probe (Bruker Corporation, Karlsruhe, Germany), observing ¹H nuclei at 600.13 MHz.

One-dimensional ¹H NMR spectra were acquired at 298 K. A pooled quality control (QC) sample, prepared from all muscle extracts samples, was used to calibrate the 90° pulse length, optimize water suppression, and monitor spectral quality during acquisition. Samples were loaded into the spectrometer in random order, and ¹H NMR spectra (128 transients, 64k data points) were recorded using the noesypr1d pulse sequence over a spectral width of ~ 20 ppm, with a mixing time of 100 ms and a relaxation delay of 4 s (Beckonert et al. 2007). Spectra were processed with TopSpin 3.7 software. All ¹H chemical shifts were referenced to the DSS-*d*₆ signal at 0.00 ppm. An exponential line broadening of 0.3 Hz was applied prior to Fourier transformation, followed by automatic zero- and first-order phasing and baseline correction.

Spectral processing, metabolite identification, and quantification were performed using Chenomx NMR Suite Professional (Chenomx Inc., Edmonton, Canada). The water signal region was excluded, and phase, baseline, and spectral alignment corrections were carried out with DSS-d₆ as the internal reference. Metabolite concentrations were determined from the ratio between the peak area of each metabolite and that of DSS-d₆, corresponding to its known concentration of 0.5 mM in each sample.

### Statistical analysis

All statistical analyses were performed using SAS (version 9.2, SAS Institute Inc., Cary, NC USA). Carcass traits were analyzed as a 2 × 2 factorial with the fixed effects of the dam´s parity (secundiparous or multiparous) and weaning strategy (early or conventional)with the dam as the experimental unit and bull as the observational unit. The slaughter group and pen (block) were used as random effect. All variables were evaluated for the normality of the residuals by the Shapiro-Wilk test and homogeneity of variance by Levene´s test. Data were analyzed using PROC MIXED, considering the effects of the weaning strategy, dam´s parity, and their respective interaction.

Meat quality traits were evaluated as repeated measurements, considering fixed effects dam´s parity, weaning strategy, maturation period and two- and three-way interactions. Covariance structures were assessed and the most appropriate structure for each variable was selected based on the corrected Akaike’s Information Criterion (AICC).

The statistical model for repeated measures over time was structured as follows:

$$\begin{aligned} \mathrm{Y} &= \upmu + \mathrm{A}_\mathrm{i} + \mathrm{WS}_\mathrm{j} + \mathrm{DP}_\mathrm{k} + \mathrm{T}_\mathrm{l} + (\mathrm{WS}\times \mathrm{DP})_{\mathrm{jk}} + (\mathrm{DP}\times \mathrm{T})_{\mathrm{kl}}\\ &\quad + (\mathrm{WS}\times \mathrm{T})_{\mathrm{jl}}+ (\mathrm{WS}\times \mathrm{DP}\times\mathrm{T})_{\mathrm{jkl}}+ \mathrm{B}_\mathrm{m} + \mathrm{F}_\mathrm{n} + \mathrm{L}_\mathrm{o} + \mathrm{S}_\mathrm{p}+ \mathrm{E}_{\mathrm{ijklmnop}} \end{aligned}$$

Where µ represents the overall mean, A_i_ is the random effect of the animal (i = 1 to 56), WS_j_ is the fixed effect of the weaning strategy (j = 1 to 2), C_k_ is the fixed effect of the dam parity (k = 1 to 2), T_l_ is the fixed effect of the time of maturation (l = 1 to 4), B_m_ is the random effect of block (m = 1 to 3), F_n_ is the random effect of the Father (*n* = 1 to 3), L_o_ is the random effect of the weaning lot (o = 1 to 5), S_p_ is the random effect of Slaughter (*p* = 1 to 4) and Eijklmnop is the residual error.

For individual measurements (Tables 2, 3 and 5), the model was:

**Table 2:** Carcass characteristics of Nelore bulls born from dams of different parities (secundiparous and multiparous) that underwent different weaning strategies (early or conventional weaning, during the 150 vs. 240 days of the previous calving season, respectively)

Item	Weaning strategy (WS)		SEM^1^	Dam parity (DP)		SEM^1^	*P* – Value^1^		
	Early	Conventional		Multiparous	Secundiparous		WS	DP	WS×DP
n	27	29	—	29	27	—	—	—	—
Age of slaughter, months	22	22	0.2	22	22	0.2	0.32	0.42	0.66
Cold carcass weight, kg	320	319	5.4	327	312	4.9	0.90	0.03	0.62
Cold carcass yield, %	55.7	55.9	0.23	55.8	55.7	0.23	0.66	0.76	0.22
Drip loss, %	2.03	2.29	0.200	2.19	2.13	0.200	0.07	0.67	0.14
Pistola Hindquarter, kg	75.4	74.2	1.06	75.6	74.0	1.08	0.39	0.29	0.90
Pistola Hindquarter, % of carcass	46.6	46.0	0.25	45.6	47.0	0.26	0.09	< 0.01	0.36
Forequarter, kg	59.4	59.9	1.15	61.8	57.5	1.31	0.73	0.01	0.39
Forequarter, % of carcass	36.6	37.0	0.26	37.1	36.4	0.27	0.33	0.09	0.47
Brisket, kg	22.5	22.3	0.48	23.2	21.6	0.52	0.79	0.03	0.32
Brisket, short ribs, and flank, % of carcass	13.8	13.8	0.21	14.0	13.6	0.22	0.96	0.29	0.58
Kidney and flank fat, kg	5.56	5.31	0.175	5.48	5.38	0.176	0.37	0.75	0.35
Hindquarter Bones, kg	14.6	14.8	0.20	14.7	14.7	0.21	0.67	0.78	0.62
Hindquarter Fat trim, kg	3.01	3.01	0.076	3.04	2.98	0.078	0.98	0.53	< 0.01
Retail cuts, % of Hindquarter	69.4	69.9	0.28	69.8	69.5	0.28	0.24	0.54	0.91

^1^SEM: Standard error of the mean; WS: Weaning effect; DP: Dam parity effect; WS×DP: Interaction between weaning strategy and dam parity

**Table 3:** Hindquarter retails cuts of carcasses from Nelore bulls born from dams of different parities (secundiparous and multiparous) that underwent different weaning strategies (early or conventional weaning, during the 150 vs. 240 days of the previous calving season, respectively)

Item	Weaning strategy (WS)		SEM^1^	Dam parity (DP)		SEM^1^	*P* – Value^1^		
	Early	Conventional		Multiparous	Secundiparous		WS	DP	WS×DP
Striploin, kg	9.96	9.94	0.169	10.02	9.89	0.176	0.93	0.62	0.61
Tenderloin, kg	1.92	1.90	0.039	1.94	1.88	0.041	0.71	0.23	0.96
Top sirloin cap, kg	1.70	1.72	0.076	1.60	1.82	0.075	0.85	0.05	0.45
Rump cap, kg	1.71	1.74	0.037	1.71	1.74	0.037	0.67	0.71	0.44
Eye of rump, kg	4.38	4.24	0.083	4.39	4.24	0.084	0.24	0.25	0.41
Knuckle, kg	5.90	5.81	0.104	5.87	5.84	0.105	0.56	0.87	0.38
Eye of round, kg	2.83	2.99	0.057	2.95	2.87	0.055	0.07	0.39	0.53
Inside round, kg	6.88	6.70	0.188	7.01	6.57	0.190	0.50	0.14	0.44
Outside round, kg	10.8	10.9	0.19	11.0	10.6	0.19	0.75	0.19	0.61
Tri-tip, kg	1.38	1.34	0.030	1.39	1.34	0.030	0.42	0.34	0.15
Hump (zebu hump), kg	6.33	5.50	0.907	5.56	6.28	0.901	0.63	0.69	0.44
Shank, kg	4.83	4.63	0.087	4.89	4.57	0.087	0.08	0.02	0.86

^1^SEM: Standard error of the mean; WS: Weaning effect; DP: Dam parity effect; WS×DP: Interaction between weaning strategy and dam parity

Y = µ + A_i_ + WS_j_ + DP_k_ + (WS×DP)_jk_ + B_l_ + F_m_ + L_n_ + S_o_+ E_ijklmno_, where µ, A_i_, WS_j_, DP_k,_ B_l_, F_m_, L_n_, S_o_ and E_ijklmno,_ have the same meanings.

The effects of period and main factor interactions were assessed by the F-test using the LSMEANS statement (SLICE option) within PROC MIXED and were considered different when *P*-values were less than 0.05 and tended to be marginally different when 0.05 < *P* ≤ 0.10.

Metabolomic data were pre-processed in the R environment (R Core Team, 2023), including imputation of missing values, removal of variables with excessive zeros, and application of Pareto scaling. The Group separation was examined through Partial Least Squares Discriminant Analysis (PLS-DA), with five-fold cross-validation (ten repetitions) and permutation testing (*n* = 3000) for model validation. Metabolite relevance was quantified using Variable Importance in Projection (VIP) scores, considering VIP > 1 as discriminant (Rohart et al. 2017). Significant metabolites were subjected to metabolic pathway enrichment analysis to identify modulated biochemical pathways. All analyses and visualizations were performed in R using the packages mixOmics (Rohart et al. 2017), tidyverse, ggplot2 (Wickham 2016), car, MetaboAnalystR, igraph (Csardi and Nepusz 2006), pheatmap (Kolde 2019), and writexl (Ooms 2023). Univariate analysis (ANOVA) was conducted for the identified discriminant metabolites, fitting weaning strategy, dam parity, and their interaction as fixed effects. Variables displaying significant interactions were sliced using Fisher’s F-test, while main effects were assessed for variables identified as significant in the multivariate analysis. Owing to the limited sample size for the metabolomics analysis, a *P* < 0.05 was adopted as the significance level in the analysis of variance.

## Results

### Carcass traits

As detailed in Carlis et al. (2025b), the primary biological criterion for slaughter was achieving an adequate subcutaneous fat thickness by ultrasound (SFTu) to ensure proper carcass finishing. According to Carlis et al. (2025b), the final SFTu prior to slaughter were no statistically different among the groups (*P* = 0.78), presenting averages of 3.6 mm for EW-MP, 3.8 mm for CW-MP, 3.8 mm for EW-SP, and 3.9 mm for CW-SP. Due to this biological criterion, combined with the logistical constraint of a maximum slaughter capacity of 14 animals per day at our experimental abattoir, the animals were harvested in sequential groups, resulting in natural variation in total days on feed. To ensure that this temporal variation did not confound the present meat quality and metabolomic analyses, the slaughter group was strictly accounted for as a random effect in all statistical models.

There was interaction WS×DP (*P* < 0.01) for hindquarter fat trim, where carcasses from EW-MP and CW-MP bulls had greater fat trim compared to those from EW-SP bulls (Table 2, Supplementary Figure S1).

However, carcasses from EW bulls tended to have smaller drip loss (*P =* 0.07) and greater (*P =* 0.09) pistola hindquarter as % of carcasses compared to carcasses from CW bulls (Table 2). Carcasses from MP had greater cold weight (*P =* 0.03), smaller pistola hindquarter as % of carcasses (*P <* 0.01), and greater brisket weights (*P* = 0.03) compared to carcasses from SP bulls. Also, carcasses from MP tended (*P* = 0.09) to have greater Forequarter as % of carcasses compared to carcasses from SP bulls (Table 2).

Carcasses from EW bulls tended to have smaller eye of round (*P =* 0.07) and greater shank (*P =* 0.08) compared to carcasses from CW bulls (Table 3). Carcasses from the MP group had a smaller (*P =* 0.05) top sirloin cap and a greater (*P =* 0.02) shank compared to carcasses from the SP (Table 3). No differences for the main effects or their interaction between cold carcass yield, pistola hindquarter (kg), Forequarter (kg), brisket, short ribs, and flank (% of carcass), kidney and flank fat, hindquarter bones, retail cuts (% of hindquarter), striploin, tenderloin, rump cap, eye of rump, knuckle, inside round, outside round, tri-tip, and hump (zebu hump) were observed.

### Meat quality and composition

There were no interactions between WS×DP or weaning strategy and dam parities and time (WS×DP×T) for meat quality parameters (Table 4). However, there was an interaction between DP×T for carcass temperature (*P =* 0.02), when carcasses from MP bulls had greater carcass temperature three hours post-slaughter compared to carcasses from SP bulls (Supplementary material, Figure S2). While the average final pH was greater in EW carcasses (*P* < 0.05; Table 4), there was a trend for an interaction between weaning strategy and time (WS×T) (*P =* 0.10), where carcasses from EW bulls showed a tendency for greater (*P* = 0.10) carcass pH compared to CW carcasses at 6, 9 and 12 h after the slaughter (Supplementary material, Figure S3). However, carcasses from MP bulls tended to have a greater temperature (*P =* 0.08) and a smaller L* (*P =* 0.10) compared to carcasses from SP bulls (Table 4). No differences for the main effects or their interaction for sarcomere, a*, b*, cooking loss, shear force, and meat composition (Tables 4 and 5).

**Table 4:** Meat quality parameters of carcasses from Nelore bulls born from dams of different parities (secundiparous and multiparous) that underwent different weaning strategies (early or conventional weaning, during the 150 vs. 240 days of the previous calving season, respectively)

Item	Weaning strategy (WS)		SEM^1^	Dam parity (DP)		SEM^2^	*P* – Value^1^					
	Early	Conventional		Multiparous	Secundiparous		WS	DP	WS×DP	WS×T	DP×T	WS×DP×T
Sarcomere, µm	1.67	1.70	0.020	1.69	1.68	0.010	0.20	0.70	0.94	—	—	—
pH	5.82	5.73	0.040	5.77	5.79	0.040	0.04	0.22	0.77	0.10	0.31	0.71
Temperature, °C	4.99	4.90	0.180	5.08	4.81	0.170	0.60	0.08	0.48	0.91	0.02	0.99
L*	44.7	45.0	0.32	44.4	45.3	0.34	0.47	0.10	0.79	0.45	0.16	0.80
a*	24.1	24.1	0.22	24.4	23.9	0.23	0.97	0.15	0.32	0.64	0.31	0.86
b*	17.6	17.7	0.19	17.7	17.6	0.20	0.69	0.81	0.37	0.52	0.15	0.99
Cooking loss, %	33.0	32.8	0.24	33.1	32.7	0.25	0.55	0.31	0.29	0.83	0.15	0.38
Shear force, N	78.4	76.5	1.97	76.4	78.5	2.06	0.50	0.51	0.19	0.74	0.52	0.59

^1^SEM: Standard error of the mean; WS: Weaning effect; DP: Dam parity effect; WS×DP: Interaction between weaning strategy and dam parity; DP×T: Interaction between dam parity and time of maturation: WS×T: Interaction between weaning strategy and time of maturation; WS×DP×T: Interaction between weaning strategy, dam parity and time of maturation

**Table 5:** Composition of the meat of carcasses from Nelore bulls born from dams of different parities (secundiparous and multiparous) that underwent different weaning strategies (early or conventional weaning, during the 150 vs. 240 days of the previous calving season, respectively)

Item^1^	Weaning strategy (WS)		SEM^2^	Dam parity (DP)		SEM^2^	*P* – value^2^		
	Early	Conventional		Multiparous	Secundiparous		WS	DP	WS×DP
N	27	29	—	29	27	—	—	—	—
Age of slaughter, months	21.8	21.9	0.18	21.9	21.8	0.18	0.32	0.42	0.66
Fat, %	2.88	3.10	0.150	2.92	3.06	0.150	0.26	0.53	0.33
Moisture, %	72.8	72.6	0.18	72.8	72.6	0.18	0.47	0.32	0.88
Protein, %	23.6	23.5	0.13	23.4	23.7	0.13	0.45	0.19	0.41
Ash, %	0.480	0.460	0.0100	0.480	0.460	0.0100	0.40	0.37	0.21
SCFA, %	1.56	1.62	0.050	1.60	1.58	0.040	0.39	0.79	0.57
Carbohydrates, %	0.450	0.410	0.0800	0.480	0.390	0.080	0.73	0.50	0.53
Water activity, %	0.990	0.990	0.0001	0.990	0.990	0.0001	0.56	0.88	0.92
Energy/ 100 g	122	123	1.4	122	124	1.4	0.47	0.23	0.36
Na, %	0.860	0.810	0.0200	0.840	0.830	0.0200	0.22	0.69	0.36

^1^SCFA: Short-Chain fatty acids.

^2^SEM: Standard error of the mean; WS: Weaning effect; DP: Dam parity effect; WS×DP: Interaction between weaning strategy and dam parity

### Meat metabolomics profile

**Fig. 3:**
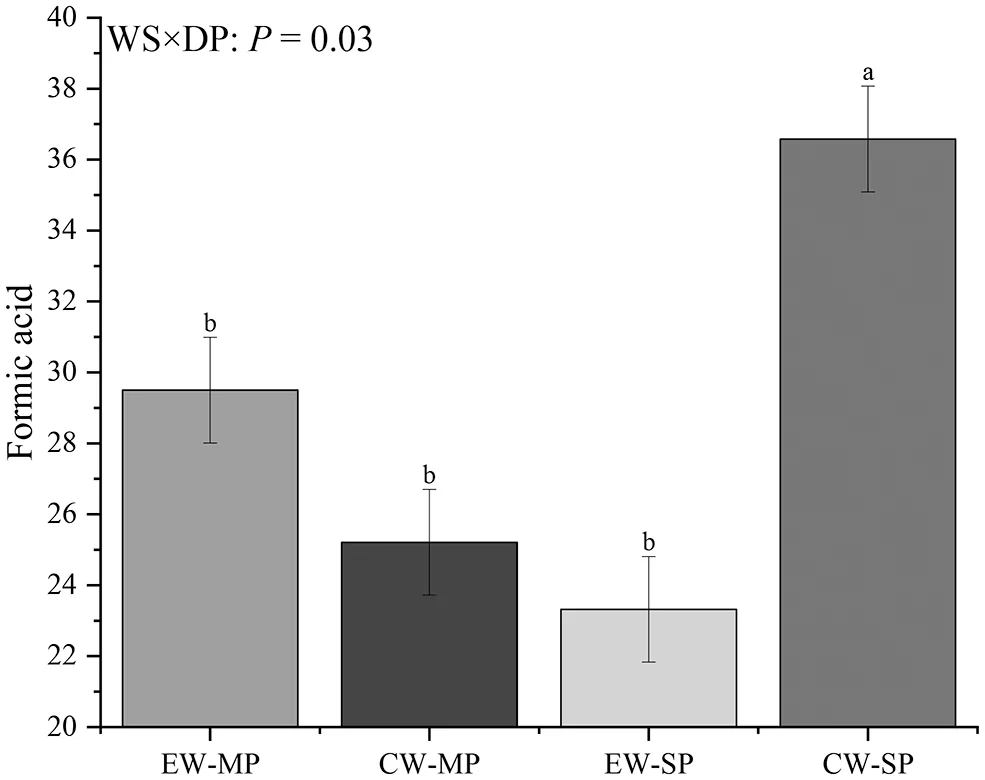
Formic acid concentration for the interaction between weaning strategy (WS; early at 150 d or 80 d of gestation and conventional at 240 d or 170 d of gestation) and Dam parity (DP; multiparous and secundiparous). Bars represent the standard error of the mean. Different lowercase letters indicate that the means differ by F-test at 5% of probability among the combinations of factors: EW-MP: Early weaning (at 150 d of lactation or 80 d of gestation; *n* = 15), in multiparous dams; CW-MP: Conventional weaning (at 240 d of lactation or 170 d of gestation; *n* = 15), in multiparous dams; EW-SP: Early weaning (at 150 d of lactation or 80 d of gestation; *n* = 13), in secundiparous dams; and CW-SP: Conventional weaning (at 240 d of lactation or 170 d of gestation; *n* = 15), in secundiparous dams

Forty-five metabolites were identified and quantified in the ^1^H-NMR spectrum of muscle. The identified metabolites included non-essential amino acids, essential amino acids, biogenic amines, organic acids, sugars, and vitamins (Supplementary Table S1). Univariate analysis revealed a significant interaction between weaning strategy (WS) and dam parity (DP) only for formic acid concentration (*P* = 0.03), where muscles from CW-SP bulls had a greater concentration compared to the other groups (Fig. 3 and Supplementary Table S1). No other differences (*P* > 0.10) were observed for the main effects or their interactions when analyzing individual metabolite concentrations (Supplementary Table S1). Therefore, multivariate approaches were employed to explore potential subtle, coordinated changes across the metabolite profile.

**Fig. 4:**
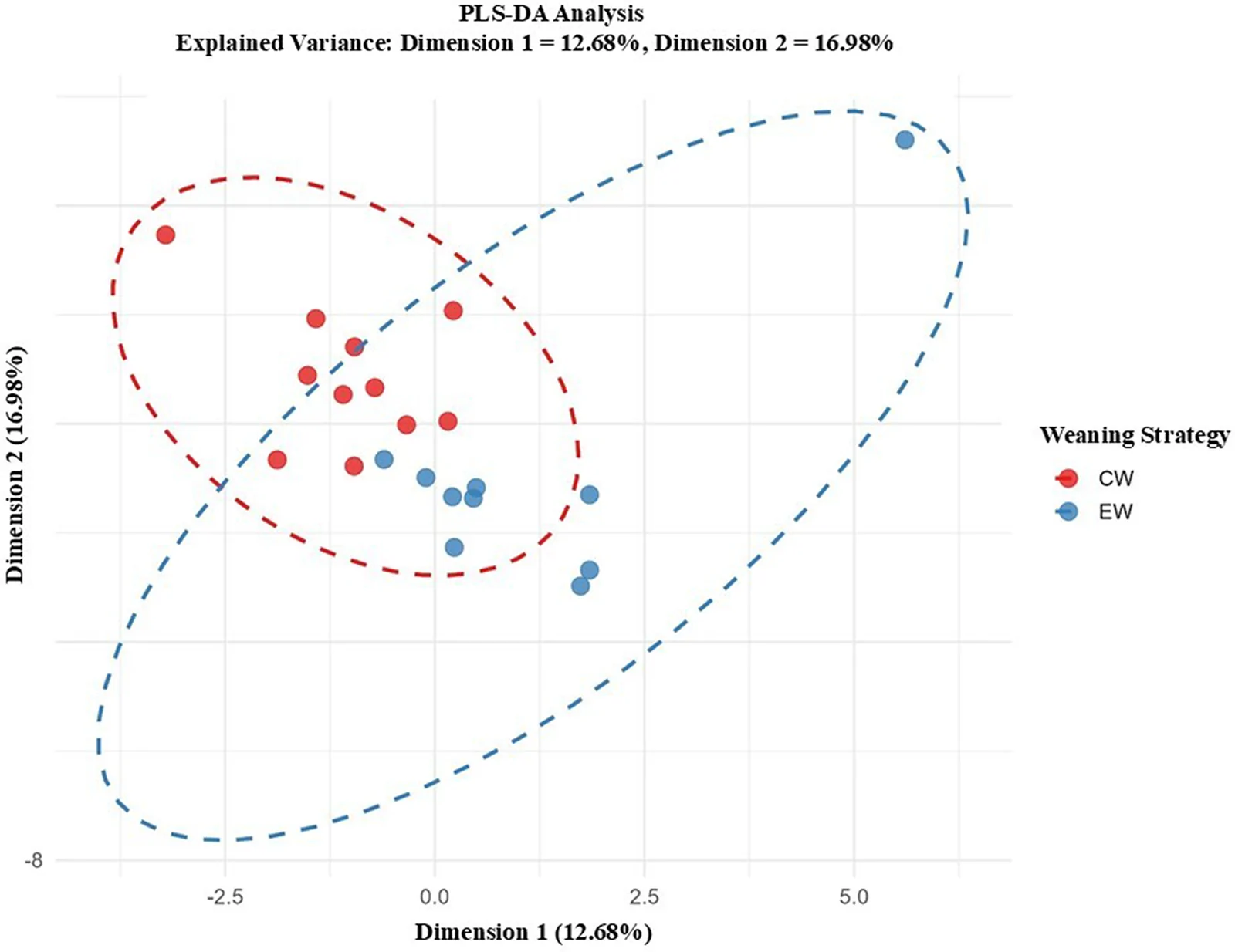
Partial least-squares discriminant analysis (PLS-DA) of the *Longissimus thoracis* muscle at slaughter for the main effect of weaning strategy (WS; early at 150 d or 80 d of gestation and conventional at 240 d or 170 d of gestation). There were two significant Components which together explain 25.46% of the total variance (Component 1 12.41% and Component 13.05%). The distribution data presented a partial overlap in the muscle metabolome data according to the treatments, suggesting differences in muscle metabolome for bulls according to weaning strategies

**Fig. 5:**
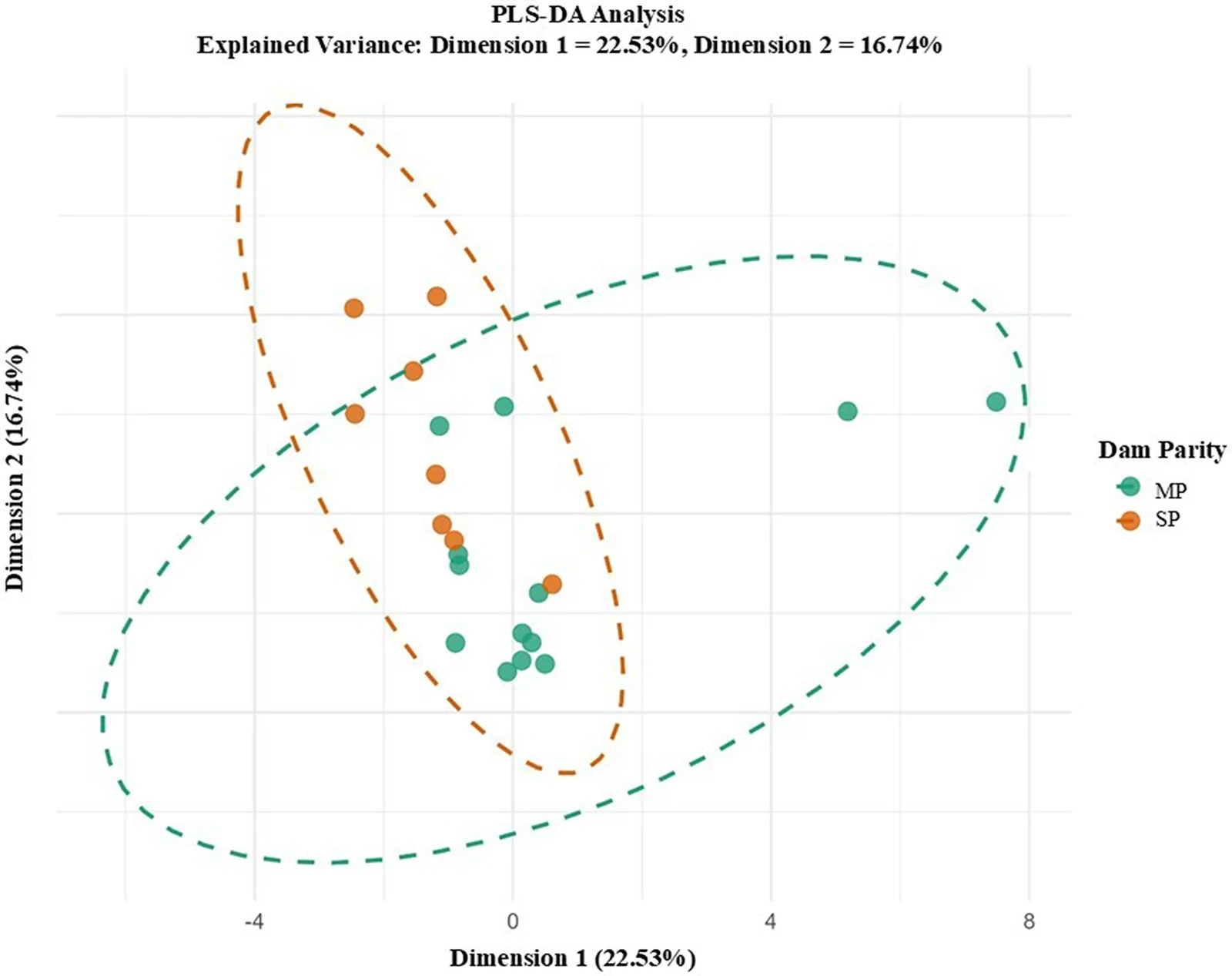
Partial least-squares discriminant analysis (PLS-DA) of the *Longissimus thoracis* muscle at slaughter for the main effect of dam parity (MP; multiparous dams and SP; secundiparous dams). There were two significant Components which together explain 37.05% of the total variance (Component 1 21.06% and Component 2: 15.99%). The distribution data presented a partial overlap in the muscle metabolome data according to the treatments, suggesting differences in muscle metabolome for bulls according to weaning strategies

PLS-DA scores plot for weaning strategy (Fig. 4) and dam parity (Fig. 5) revealed considerable overlap between groups. This substantial overlap implies that rather than producing a completely distinct or divergent global metabotype, the maternal interventions subtly reprogrammed specific metabolic nodes and targeted pathways within the offspring’s muscle. However, the models captured a portion of the variance (Weaning Strategy: Dimension 1 = 12.68%, Dimension 2 = 16.98%; Dam Parity: Dimension 1 = 22.53%, Dimension 2 = 16.74%) and permutation testing confirmed model validity (Accuracy = 0.684; R^2^ = 0.989; Q^2^ = 0.067).

**Fig. 6:**
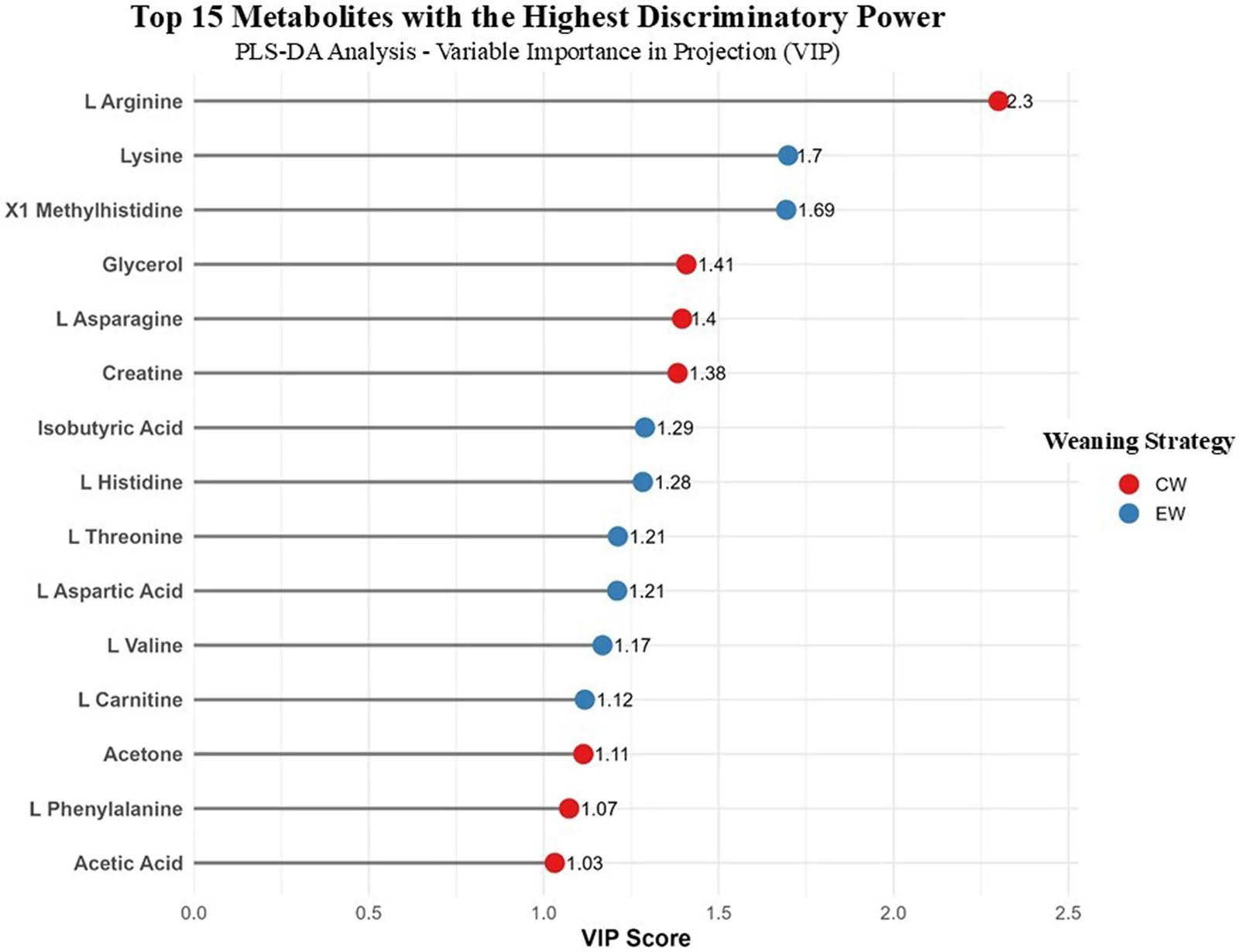
Variable importance in projection **(**VIP) plot of metabolites differentially abundant in the *Longissimus thoracis* muscle at slaughter for the main effect of weaning strategy (WS; early at 150 d or 80 d of gestation and conventional at 240 d or 170 d of gestation)

**Fig. 7:**
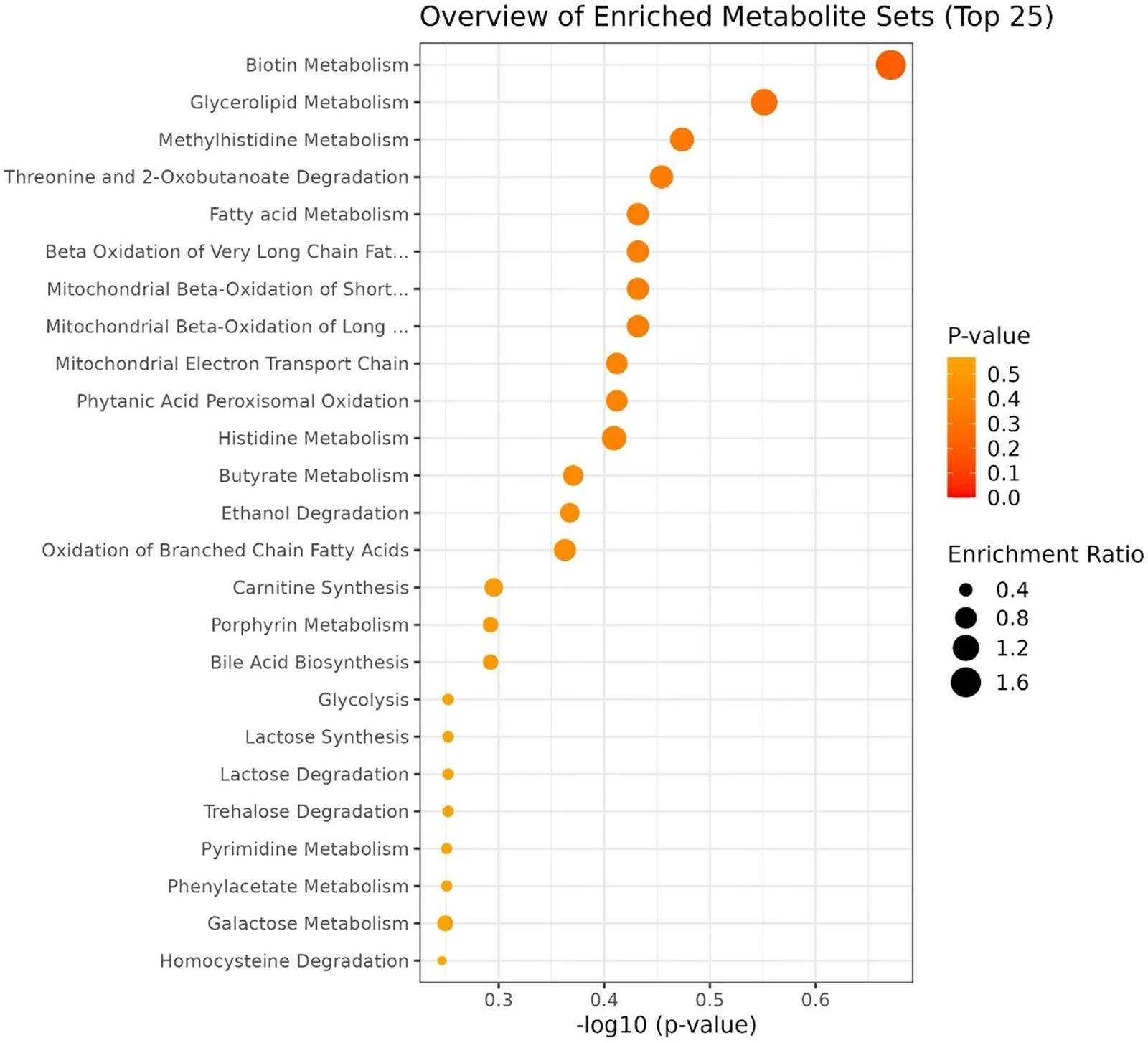
Functional enrichment analysis of metabolites in the *Longissimus thoracis* muscle at slaughter for the main effect of weaning strategy (WS; early at 150 d or 80 d of gestation and conventional at 240 d or 170 d of gestation)

To identify the metabolites most influential in discriminating between weaning strategies, Variable Importance in Projection (VIP) scores were examined (Fig. 6). Amino acids and derivatives associated with protein synthesis or energy status, specifically lysine, L-asparagine, creatine, L-histidine, L-threonine, L-aspartic acid, and L-phenylalanine, along with the energy substrate glycerol, were identified as key discriminants between groups (VIP > 1). Notably, these metabolites exhibited greater concentrations in the muscle tissue of EW bulls. Conversely, L-arginine, 1-methylhistidine (a marker of myofibrillar protein breakdown), L-valine, and metabolites linked to fatty acid or BCAA catabolism (such as isobutyric acid and L-carnitine) were also significant for group discrimination (VIP > 1), presenting elevated levels in the muscle of CW bulls. Metabolite set enrichment analysis was performed to identify biological pathways potentially altered by the interventions. Comparing EW vs. CW groups (Fig. 7), pathways showing significant enrichment (*P* < 0.05, indicated by -log10(p-value) > 1.3) included Biotin Metabolism, Glycerolipid Metabolism, and Methylhistidine Metabolism, suggesting impacts on one-carbon metabolism, lipid turnover, and protein degradation dynamics.

**Fig. 8:**
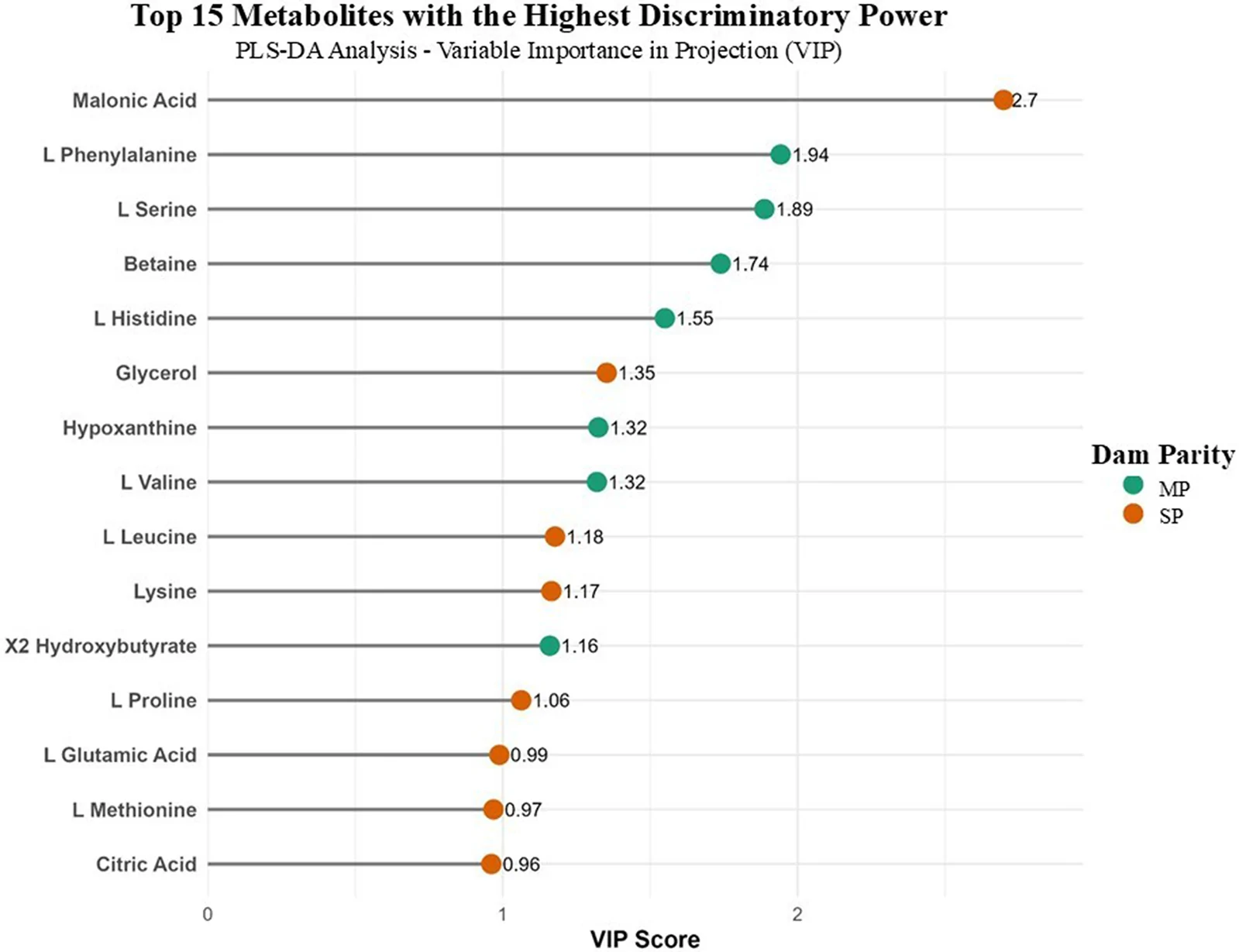
Variable importance in projection **(**VIP) plot of metabolites differentially abundant in the *Longissimus thoracis* muscle at slaughter for the main effect of dam parity (MP; multiparous dams and SP; secundiparous dams)

When comparing dam parities (Fig. 8), muscles from MP offspring amino acids like L-Phenylalanine, L-Serine, L-Histidine, L-Valine, and Lysine, as well as Hypoxanthine and 2-Hydroxybutyrate, were identified as key discriminants between groups. These metabolites exhibited greater concentration in the muscle tissue of MP offspring. In contrast, Malonic Acid (involved in fatty acid synthesis), Glycerol, Betaine, and several amino acids, including L-Leucine, L-Proline, L-Glutamic Acid, and L-Methionine, were also significant for group discrimination (VIP > 1), presenting elevated levels in the muscle of SP offspring.

**Fig. 9:**
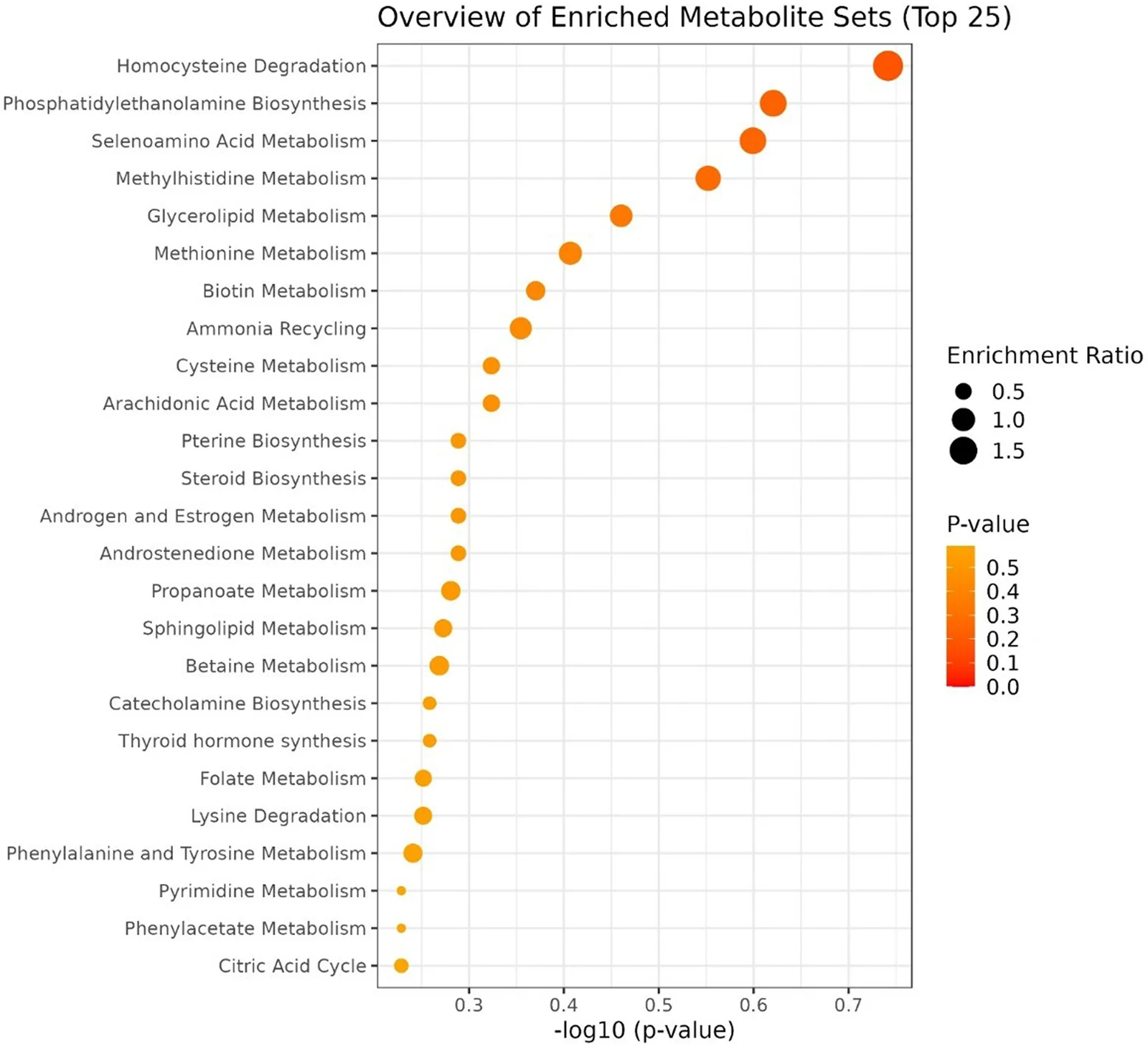
Functional enrichment analysis of metabolites in the *Longissimus thoracis* muscle at slaughter for the main effect of dam parity (MP; multiparous dams and SP; secundiparous dams)

The comparison between dam parities (MP vs. SP, Fig. 9) revealed significant enrichment (*P* < 0.05) in pathways such as Homocysteine Degradation, Phosphatidylethanolamine Biosynthesis, Selenoamino Acid Metabolism, and Methylhistidine Metabolism, highlighting differences in amino acid processing, membrane lipid synthesis, and potentially antioxidant defense related to maternal parity.

## Discussion

The interaction observed for hindquarter fat trim, where EW-SP carcasses exhibited less fat-trim than EW-MP and CW-MP groups, requires interpretation considering findings of Carlis et al. (2025a). Our previous results reported a tendency for lower SFT specifically in the CW-SP group and a significant interaction for muscle fiber number, with CW-SP having fewer fibers compared to CW-MP and EW-MP, while EW-SP was intermediate, using the same animals in this study. Despite the subcutaneous fat thickness at slaughter did not differ among treatments, which was expected as an SFT of 3 mm was utilized as the biological target criterion for slaughter selection. Taken together, the reduced fat trim in EW-SP might reflect the combination of adequate SFT and potentially denser musculature (unlike CW-SP), resulting in a leaner hindquarter cut compared to MP groups. It is well documented that greater fat trimming reflects greater carcass adiposity (Brigida et al. 2018). Carcasses from animals with longer finishing periods, greater body-condition scores, and greater subcutaneous fat thickness typically exhibit the fattest coverage on the carcass (May et al. 1992; Apple et al. 1999).

While altered muscle cellularity provides a partial explanation, the reduced fat trimming observed in EW-SP carcasses must also be interpreted through the lens of adipogenesis timing and finishing dynamics. Fat depots mature chronologically, with intermuscular fat, a primary component of hindquarter trim, generally developing alongside or slightly preceding subcutaneous fat. Because bulls in this study were slaughtered upon reaching a uniform target of 3 mm subcutaneous fat thickness (Carlis et al. 2025a), the smaller trim fat in EW-SP carcasses may suggest an altered trajectory of lipid partitioning. Specifically, EW-SP bulls achieved the required subcutaneous fat cover for slaughter without simultaneously accumulating intermuscular fat. This aligns with the performance data from Carlis et al. (2025a), where EW-SP bulls demonstrated an efficient compensatory growth trajectory following the weaning intervention, achieving adequate slaughter endpoints without partitioning excessive energy into dissectible trim fat, unlike their multiparous counterparts, who accumulated greater overall carcass mass and fat depots.

Furthermore, the isolated, yet marked, interaction observed for formic acid concentration, which was significantly elevated in the CW-SP bulls, warrants specific consideration. For years, the significance of formate in one-carbon metabolism (OCM) has been underappreciated, despite its critical role during developmental stages (MacMillan et al. 2016). Formate is primarily produced by mitochondria and incorporated into the cytosolic tetrahydrofolate pool, serving as an essential one-carbon unit for three processes: purine synthesis, thymidylate synthesis, and transmethylation reactions (MacMillan et al. 2016; Ducker and Rabinowitz 2017). Recent evidence highlights that OCM directly interfaces with mitochondrial bioenergetics, acting as a central integrator that links maternal nutrient supply to the offspring’s metabolomic phenotype. Mitochondria respond to OCM-derived substrates while simultaneously regulating OCM flux through formate production and redox balance. Given that the CW-SP group originated from the most nutritionally constrained maternal environment (secundiparous dams subjected to prolonged lactation), the accumulation of formate in their muscle tissue likely represents a persistent biochemical signature of fetal adaptation. Specifically, this accumulation suggests a compensatory upregulation of mitochondrial formate production aiming to fuel cytosolic OCM. This pathway facilitates the generation of S-adenosylmethionine (SAM), the universal methyl donor for epigenetic regulation, and supports the biosynthesis of nucleotides and amino acids required for cellular proliferation and myofiber remodeling during the feedlot phase (Cracco et al. 2023). Therefore, the elevated formate concentration potentially indicates a metabolic prioritization of substrate availability for anabolic recovery. However, consistent with the marginal phenotypic outcomes observed, this compensatory molecular and mitochondrial shift was ultimately insufficient to induce significant macroscopic alterations in carcass conformation or fully overcome the developmental constraints imposed in utero.

Selective breeding for improved beef carcass traits has prioritized high-value regions such as the hindquarter, which exhibits moderate heritability and thus responds readily to genetic pressure (Climaco et al. 2011). Indeed, hindquarter yield closely correlates with both average daily gain and cold carcass weight (Pabiou et al. 2009). In this context, enhanced maternal nutrition during late gestation could bolster fetal skeletal muscle development (Meneses et al. 2022), promoting the growth of carcass cuts that are already genetically selected to exhibit greater muscle hypertrophy.

An interesting observation was the tendency for the EW group to yield a greater proportion of the pistola hindquarter and shank relative to carcass weight compared to CW. These findings align with (Greenwood et al. 2009), suggesting that EW programming potentially influenced carcass conformation, although these phenotypic shifts remain subtle. Intriguingly, this occurred despite a tendency for the EW bulls to have smaller ribeye and have significantly smaller muscle fiber diameters at slaughter, as reported in Carlis et al. (2025). This apparent discrepancy may highlight that REA alone may not fully capture the programming effects on overall muscularity (Greenwood et al. 2009). The enhanced relative hindquarter yield is likely driven by altered muscle architecture and density. This mechanism is supported by direct quantitative histology performed on these exact same animals and published in our companion paper (Carlis et al. 2025a). In that study, comprehensive morphometric analysis revealed that EW bulls exhibited significantly smaller muscle fiber cross-sectional areas and diameters, while maintaining total fiber numbers compared to specific CW groups. Therefore, the coexistence of a potentially improved cut yield with smaller REA is not merely hypothetical but grounded in histological evidence of altered fiber density and differential growth favoring other hindquarter muscles.

Indeed, the metabolomic profile presented herein provides a mechanistic explanation for this improved conformation. Muscles from EW bulls displayed greater concentrations of anabolic substrates, specifically creatine and lysine, alongside a significant enrichment of Biotin Metabolism and Histidine Metabolism pathways. Beyond its role in energy homeostasis, creatine acts as a compatible osmolyte; its accumulation enhances cellular hydration, a potent anabolic signal that stimulates myofibrillar protein synthesis (Häussinger et al. 1993; Kreider et al. 2017). Furthermore, the prominence of biotin metabolism is biologically consistent with high-growth phenotypes, as biotin serves as an essential cofactor for carboxylases governing energy production and the metabolism of BCAAs, which are critical for muscle accretion (Riveron-Negrete and Fernandez-Mejia, 2016). Concurrently, the activation of histidine and methylhistidine metabolism suggests a state of active protein turnover and remodeling. This metabolic signature aligns with the upregulated mRNA expression of *EIF4E* and a tendency for greater *MTOR* expression (Han et al. 2008), observed in these same animals Carlis et al. (2025a).

Therefore, one interpretation is that EW programming resulted in muscles that, while potentially having smaller fibers at the specific slaughter endpoint, maintained a greater state of anabolic signaling and metabolic activity throughout the finishing phase. This sustained potential for hypertrophy, reflected in both the gene expression and metabolomic data, could contribute to greater overall muscle mass deposition in the hindquarter relative to the carcass frame, even if not fully realized as larger individual fiber diameters or a larger REA measurement between the 12th and 13th ribs. The tendency for an increased shank yield, representing a more distal and perhaps later-maturing muscle group (Gaili and Nour 1980). This might further support the idea of altered developmental trajectories or sustained growth potential in EW bulls. This contrasts with potential metabolic thriftiness adaptations in CW animals suggested by their greater concentration of metabolites for lipid oxidation markers like carnitine (Muroya et al. 2021). Further investigation, integrating detailed muscle composition and fiber type analysis across different hindquarter muscles, could fully elucidate these complex programming effects.

In this study, EW at 80 days of gestation effectively ceased lactational nutrient requirements on the dam and likely redirected these nutrients toward fetal development, enhancing myogenic proliferation and increasing muscle fiber number as suggested by Du et al. (2017). As a result, bulls born to EW dams suggested a potential improvement in carcass conformation, indicated by a tendency for greater hindquarter and shank yields compared to CW bulls. These outcomes correspond with the smaller muscle fiber cross-sectional area and diameter observed by Carlis et al. (2025a), which reflect greater fiber density and overall muscling (Du et al. 2010a, b, 2017). Conversely, probably reduced fetal nutrient supply and slower pre‐weaning growth rate in CW and SP bulls (Carlis et al. 2025b) may predispose to uneven muscle development, as SP carcasses displayed greater top sirloin cap yields than their MP peers.

Carcass size, fat content, proteolysis, and pre-slaughter handling are the primary determinants of ultimate pH and the rate of postmortem pH decline (Leygonie et al. 2012; Lancaster et al. 2020; Sullivan et al. 2022). Enhanced proteolysis during chilling releases greater amounts of water and buffering compounds through the deamination of amino acids, which can reduce the rate of carcass pH drop (Leygonie et al. 2012). The greater ultimate pH and tendency for smaller drip loss in EW carcasses, therefore, suggest improved water holding capacity. This may relate to the muscle’s metabolic state or post-mortem processes. Our metabolomic analysis revealed a greater concentration of histidine in the EW muscle. Evidence indicates histidine works as a precursor for carnosine and anserine, dipeptides exhibiting potent intracellular buffering capacity, while concurrently functioning as a biomarker for type II fast-twitch muscle fibers (Abe 2000; Seong et al. 2015). The greater concentration of histidine in EW groups may thus explain both the greater meat pH and the potential for improved water retention properties in these carcasses (Abe 2000). Similarly, 3-methylhistidine accumulation directly correlates with myofibrillar fragmentation, as this post-translationally modified residue occurs in actin and myosin heavy chains. (Wohlt et al. 1982). Consequently, the greater methylhistidine concentration observed in CW musculature suggests enhanced proteolytic disruption of myofibrillar integrity (Gopinath and Kitts 1984), potentially contributing to their tendency for greater drip loss.

It is important to contextualize the marginally significant outcomes observed for specific carcass traits. A retrospective power analysis indicated that the sample size utilized (*n* = 28 per main effect) possessed 80% power (at α = 0.05) to detect a minimum detectable difference of 0.15 percentage points for drip loss and 2.64 percentage points for pistola hindquarter yield. The actual differences observed between the EW and CW groups (0.26 and 0.60 percentage points, respectively) reflect subtle phenotypic shifts, with the pistola hindquarter yield falling considerably below its critical statistical threshold. Nonetheless, while these specific phenotypic measurements did not strictly cross the *P* < 0.05 threshold, their directional shifts aligned coherently with the robust and highly significant alterations observed at the molecular level. This suggests that the subtle, long-term physiological programming induced by early weaning may have modulated altered muscle metabolic pathways and buffering capacity, even if the gross phenotypic expression remained marginal at the chosen statistical threshold.

The distinct muscle metabotype identified via PLS-DA and VIP scores align compellingly with gene expression patterns reported by Carlis et al. (2025a). The EW profile featured a greater relative abundance of amino acids and derivatives linked to protein turnover and energy status, such as lysine, asparagine, creatinine, and histidine (Don et al. 2025). This metabolic signature corresponds coherently with the upregulated EIF4E and tendencies for greater *MTOR* and *PPARA* mRNA expression observed in these same bulls as in Carlis et al. (2025a), indicators of enhanced protein synthesis potential. This suggests programming effects of EW may have fostered a muscle environment conducive to sustained anabolism during feedlot finishing.

In contrast, the CW profile showed greater concentration for metabolites associated with lipid oxidation (carnitine) and BCAA catabolism, isobutyrate, valine, (Rastello et al. 2025; Ye et al. 2025), potentially reflecting metabolic thriftiness programmed by relative nutrient constraint in utero (Hales and Barker 1992; Cracco et al. 2023). These findings align with reports associating elevated free amino acid concentrations with rapidly growing cattle (Gómez et al. 2022), however, the comparable growth rates between EW and CW bulls in this study likely explain the absence of significant shear force differences. Notably, the elevated concentration of 1-methylhistidine in CW musculature further differentiates the groups, indicating potentially greater preferential protein degradation relative to synthesis in these animals (Wohlt et al. 1982; Gopinath and Kitts 1984). This pattern contrasts sharply with the EW bulls, where the combined metabolic and gene expression signatures suggest sustained muscular hypertrophy potential through the finishing phase, consistent with their tendency for an enhanced relative hindquarter yield.

Collectively, these metabotype differences imply that CW bulls developed metabolic adaptations stemming from gestational conditions, potentially favoring lipid utilization over extensive muscle protein accretion, although these adaptations appear primarily confined to the muscle metabotype level without systemic consequences detected. Furthermore, the elevated concentration of glycerol observed in EW bull muscle, together with the enrichment of glycerolipid metabolism in the pathway analysis, may reflect potentially increased triglyceride turnover and lipolysis within intramuscular adipocytes (Cônsolo et al. 2022; Muroya et al. 2022a). This suggests that EW might promote a greater rate of lipid mobilization, possibly correlating with energy production to support the observed anabolic signaling. However, as proximate composition analyses did not reveal differences in intramuscular fat deposition, these metabolomic shifts related to lipid dynamics appear subtle at the endpoint measured.

These interconnected pathways indicate that EW exerts coordinated effects on both lipid and protein metabolism, highlighted by the enrichment of biotin metabolism (linked to one-carbon metabolism and potentially lipogenesis) and glycerolipid metabolism pathways in the WS pathway analysis. While these findings point to clear metabolic programming, further research integrating multi-omics data over time is warranted to fully elucidate how maternal nutrition and weaning strategies modulate the long-term trajectory of muscle development and composition in Nelore cattle.

Carcass cut yield is primarily driven by cold carcass weight (Brigida et al. 2018), accordingly, bulls born from MP dams showed heavier carcasses and exhibited greater forequarter and brisket weights, leading to increased shank weights compared to bulls born from SP, despite having a smaller relative hindquarter yield. Lancaster et al. (2020), reported that, in carcasses differing by approximately 96 kg, larger carcasses displayed a slower decline in internal temperature during chilling, although ultimate temperatures did not differ. Consistent with these findings, MP bull carcasses in the current trial retained greater temperatures at three hours postmortem than SP carcasses.

Enhanced protein turnover, characterized by elevated synthesis and degradation rates of muscle proteins, is frequently observed in carcasses with greater muscularity (Garlick et al. 1973; Gómez et al. 2022). Accordingly, the altered amino acid profile and increased 2-hydroxybutyrate abundance in SP bull musculature reflect heightened amino acid catabolism for energy production (Imaz et al. 2022; Li et al. 2022), potentially attributable to limited glycerol availability. This metabolic shift corresponds with the reduced muscling in SP bulls and may explain elevated glutamic acid levels, a key mediator in urea cycling and nitric oxide synthesis (Muroya et al. 2022b).

Conversely, MP bulls demonstrated superior carcass weights and forequarter masses alongside a metabotype conducive to muscular hypertrophy. Their elevated concentration of essential amino acids (lysine, phenylalanine, valine) may suggest activation of mTOR-mediated protein synthesis and suppression of ubiquitin ligase pathways (Sadri et al. 2016; Ferreira and Duarte 2022). Furthermore, heightened concentration of serine may indicate enhanced substrates for hepatic gluconeogenesis (Cônsolo et al. 2022; Polizel et al. 2024), enabling energy production without muscle protein mobilization. Concurrently, the greater histidine scores may reduce oxidative damage through carnosine-mediated mechanisms, while serine potentially supports membrane integrity via phosphatidylserine synthesis (Yi et al. 2025). These collective metabolic adaptations underpin the superior muscularity observed in MP carcasses.

Meat lightness (L*) is influenced by several factors, including myoglobin state, intramuscular fat content, post-mortem pH and temperature decline, and animal age (Filipčík et al. 2009; Lancaster et al. 2020; Leygonie et al. 2012). In this study, carcasses from MP dams tended to have lower L* values compared to those from SP dams. This could partially be attributed to their greater carcass mass; larger myofiber diameters, often associated with heavier carcasses, can reduce light scattering and result in lower L* readings (Han et al. 2024).

Furthermore, the observed tendency for lower L* aligns with the distinct muscle metabotype. MP musculature exhibited significantly greater concentration of histidine. Histidine serves as a precursor for the dipeptide’s carnosine and anserine, which possess potent intracellular buffering capacity and antioxidant properties that help stabilize meat color (Abe 2000; Seong et al. 2015). Enhanced buffering capacity, indicated by the elevated histidine levels in the MP group, might attenuate the rate or extent of post-mortem glycolysis (Sandberg 2017). A slower pH decline or slightly different final pH profile can influence myoglobin denaturation and water distribution within the muscle, consequently impacting light reflectance. Therefore, the greater relative abundance of histidine in MP muscle, potentially leading to greater buffering and antioxidant capacity, likely contributes to the observed tendency for lower L* values in this group compared to SP offspring.

Collectively, these enriched metabolite sets reveal that DP modulates both amino acid and lipid metabolic networks in offspring muscle, underpinning the divergent carcass phenotypes observed between MP and SP bulls. The DP factor exhibited pronounced activation of homocysteine degradation, phosphatidylethanolamine biosynthesis, selenoamino acid and methylhistidine metabolism, as well as glycerolipid, methionine, and biotin pathways, each of which supports enhanced protein synthesis, antioxidant capacity, membrane integrity, and energy provision via gluconeogenesis (Gómez et al. 2022). Concurrently, the up-regulation of ammonia recycling and cysteine metabolism further suggests a finely tuned nitrogen balance favoring muscle accretion.

While overall meat tenderness and composition were largely unaffected, the distinct muscle metabotype revealed by this study, particularly when interpreted alongside performance and gene expression data Carlis et al. (2025a), underscore the significant and lasting biological impact of both the early weaning strategy and dam parity on muscle development and metabolism.

Future investigations utilizing larger cohorts or alternative growth endpoints are warranted to definitively confirm the extent of these macroscopic conformation changes.

## Conclusions

In conclusion, early weaning to the dam is a fetal programming stimulus that significantly reshapes the muscle metabolome of Nelore bull offspring, particularly altering pathways involved in lipogenesis, phospholipid synthesis, one-carbon metabolism, and amino acid utilization. These metabolic shifts likely contribute to the potential enhancement in relative hindquarter muscularity suggested in EW bulls, achieved without compromising measured meat quality attributes such as tenderness and color. Offspring of multiparous dams consistently showed superior absolute carcass weights and greater yields of specific cuts, such as the forequarter and shank, highlighting the persistent influence of maternal parity. These results suggest that weaning management can modulate fetal programming effects on muscle metabolism and carcass traits, with outcomes potentially influenced by dam parity.

## Supplementary Information

Below is the link to the electronic supplementary material.

Supplementary Material 1

## Data Availability

Data will be made available on request.
